# A comparative evaluation of child health care in China using multicriteria decision analysis methods

**DOI:** 10.1186/s12913-023-10204-4

**Published:** 2023-11-07

**Authors:** Miao Wu, Qian Liu, Zhengye Wang

**Affiliations:** 1 College of Medical Engineering and Technology, Xinjiang Medical University, Urumqi, 830011 China; 2 Basic Medical College, Xinjiang Medical University, Urumqi, 830011 China

**Keywords:** Comparative evaluation, Child health care, Multicriteria decision analysis, Weighted TOPSIS, Weighted RSR, Fuzzy comprehensive evaluation

## Abstract

Child health is an important public health issue in China and the Chinese government always attached great importance to child health care. With the implementation of a series of medical and health reforms in China in recent decades, the status of child health improved year by year.

**Objectives **This study aims to comprehensively evaluate if the measures implemented in the medical and health reforms effectively promoted the development of Chinese child health care in recent years and provide theoretical support for future decision-making on the policies of child health care in China.

**Methods **A total of six indicators were selected from the China Health Statistics Yearbook. Based on the multi-criteria decision analysis (MCDA) algorithm, three different evaluation methods were applied in the study, which are the weighted technique for order preference by similarity to an ideal solution (TOPSIS) method, the weighted rank-sum ratio (RSR) method, and the fuzzy comprehensive evaluation (FCE). Each indicator's weight was calculated by the entropy weight methods objectively. The sensitivity analysis was conducted to validate the stability and accuracy of the rank results.

**Results **The results indicated that the rank values of each year’s child health care calculated by the different evaluation methods were not exactly the same, but the overall trend is consistent which is that child health care in China improved year by year from 2000 to 2020. The top 5 were ranked from 2016-2020 and the bottom 5 were ranked from 2000-2004.

**Conclusions **The results indicated that the policies and measures implemented in the medical and health reforms, as well as improved sanitation conditions, availability of healthy food and water, etc., have jointly promoted the development of child health care in China in the past 20 years, providing a scientific theoretical basis for future policy-making to promote child health care.

## Introduction

Child health care (CHC) is one of the most important factors for the growth of the child and is always a top priority issue in people’s health care because children are the future of a nation and their health is vital to a nation’s future development [1, 2]. As the most populous nation in the world, China always attaches great importance to maternal and CHC. Since the People’s Republic of China was founded in 1949, the central government not only mandated the development of the gynecology and pediatric department in general hospitals but also established an independent maternal and child healthcare (MCH) institution system to implement public health duties while at the same time carrying out basic medical services that are closely related to the health of women and children [3]. With the efforts of several generations, China has established a large sound MCH system with improved service delivery and health status, benefiting over 2/3 of the total population and beyond. In particular, the under-five mortality rate dropped from 210.7 to 7.8 deaths per 1,000 live births during the past 70 years. And the maternal mortality rate dropped from 1500 to 17.8 over the same period [4, 5].

In September 2000, global leaders, including Chinese leaders, gathered at the United Nations assembly and adopted a resolution on the Millennium Development Goals (MDG) which were 8 goals that United Nations Member States tried to achieve before 2015. Among the main objectives are a 2/3 reduction in child mortality in the under-fives (MDG 4) and a 3/4 reduction in maternal mortality (MDG 5) [6]. Remarkable progress in achieving the MDG has been made during this period [7]. In 2009, China launched a comprehensive health reform, as part of the central government’s plan to improve its healthcare system, aiming to provide universal coverage of essential health services for all Chinese citizens by 2020 [8]. This health reform can be broadly classified into 2 phases: the first one was from 2009 to 2011, and the other was from 2012 onward [9]. The first phase emphasized financial investment and focused on increasing financial investment to expand insurance coverage and build infrastructure [9]. As a consequence, the proportion of total health expenditure to gross domestic product (GDP) increased from 4.55 in 2008 to 6.64 in 2019 [10]. The second phase prioritized the transformation of resources into effective services through systemic healthcare delivery reform [9]. Recognizing the inadequacies of the first phase, the government moved to address the systemic causes of the inefficient healthcare delivery system, including altering provider payment and pricing incentives, restructuring macro-governance, and reforming the health delivery system. Public hospital reform and a primary-healthcare-based integrated delivery system consisted of China’s healthcare delivery transformation. Recognizing the complexity of delivery reform, the central government issued general guidelines and, except for the Zero-Markup Drup Policy, encouraged local governments to innovate and experiment with models within their institutional context [9]. With the past decade’s effort, China has made substantial progress in improving equal access to care and enhancing financial protection, especially for people of a lower socioeconomic status [9]. In 2019, China also issued the Healthy China Action Plan (HCAP) which is a new guideline to implement the country’s initiative to improve health throughout the lifespan. Given the particular importance of childhood and adolescence for overall lifelong health, the HCAP aims to foster child and adolescent health and well-being through a series of steps and programs [11]. Furthermore, To arrest the falling birth rate, in recent years, the Chinese government has ramped up efforts to encourage families to have more children with the implementation of the policy on encouraging childbirth in China [12]. As a consequence, the child population in China might become larger than before, and any improvement in CHC services or policies will benefit hundreds of millions of children in China. The policies implemented in China may have little bias in different regions due to the different conditions and environments, however, the overall direction of policies is consistent.

Under the above background, we try to use multi-criteria decision-making (MCDM) methods to scientifically evaluate if the measures of China’s medical and health reform promoted the CHC status in recent years and provide theoretical support for future decision-making on the policies of CHC. MCDM is a method to support decision-making, by exploring the balance between the pros and cons of different alternatives [13]. And it was used widely in many fields which will be discussed in the Literature review part. In this study, 3 methods of MCDM which are the weighted technique for order preference by similarity to an ideal solution (TOPSIS), the weighted rank-sum ratio (RSR) method, and the fuzzy comprehensive evaluation (FCE) method, have been applied to comprehensively evaluate the status of CHC in China during 2000-2020. A total of 6 evaluation indicators and corresponding values were selected from the China Health Statistic Yearbook. To the best of our knowledge, few studies focused on the evaluation of the Chinese child healthcare state in recent years.

The remaining part of this paper consists of the following 5 parts: Literature Review, Data and Methods, Results, Discussion, and Conclusions.

## Literature review

MCDM, also known as Multi-Criteria Decision Analysis the method that supports decision-makers faced with evaluating alternatives by taking into account multiple criteria in an explicit manner [14, 15]. It has been widely applied in the public sector as well as in private-sector decisions on agriculture resource management, immigration, education, transport, investment, environment, defense, health care, etc. [16]. The application in the medicine and healthcare field has been booming since the 2000s [17]. MCDM approaches can be classified broadly into 3 categories: value measurement models, outranking models, and goal, aspiration, or reference-level models [18]. Inspired by the previous study [19], we use outranking models to rank the CHC state of each year in China from 2000 to 2020. The common ranking methods of MCDA include TOPSIS [20], RSR [21], weighted sum method (WSM) [22], Vsekriterijumsko Kompromisno Rangiranje (VIKOR) [23], Elimination et Choice Translating Reality (ELECTRE) [24], Analytic Hierarchy Process (AHP) [25], etc.

The TOPSIS is a classical and simple method in MCDA that was first introduced by Hwang & Yoon in 1981 [26], then a series of improvements of the method has been developed and applied in the various MCDA issues [27–29]. These studies indicated that though the traditional TOPSIS method could be used in many MCDA issues, it was not sufficient to solve some sophisticated cases in real-world situations which are involved uncertainty, subjectivity, and incomplete information [30]. Cioca, et al. [31] suggest that the combined approach relying upon TOPSIS and other MCDA methods such as AHP could be more reliable and effective in solving the problem. Cao, et al. [32] objectively evaluated the conditions for scale management suitability by applying the entropy-TOPSIS method. The research gave a scientific reference for the rational utilization of land resources and land use policymaking. Jyotdeep Singh, et al. [33] applied a hybrid approach of fuzzy TOPSIS and grey relation analysis (GRA) method to strategically rank store location based on the multi-criteria. Yu, et al. [34] proposed an integrated evaluation approach to select the best suppliers by incorporating decision makers’ risk attitudes using the ANN, AHP, and TOPSIS methods. The results show that the proposed integrated method is effective and efficient.

RSR is another common evaluation method in MCDA, which was originally proposed by a Chinese professor named Tian Fengdiao in 1993 [35]. It integrates the strongpoints of classical parametric estimations and modern nonparametric estimations [36]. Due to its flexibility and outstanding performance, RSR has been widely used in the medical health field and others in recent years. Wang, et al. [37] applied RSR to the evaluation of feeding practices behaviors, and their association with infant health risks in rural Lhasa, Tibet. Wu, et al. [38] applied RSR and the data envelopment method to evaluate the medical service efficiency in Traditional Chinese Medicine (TCM) hospitals and provide references for making relevant policies scientifically. Tian, et al. [39] evaluated the overall indoor air quality by integrating air change effectiveness and contaminant removal effectiveness by using multi-indicator methods, including RSR, TOPSIS, and Z-score methods. Zhu, et al. [40] used RSR to determine the optimal parameters in their study of a biclustering algorithm in the spontaneous reporting system of China.

Previous studies indicated a variety of techniques could be used for calculating criteria’ weights in MCDA. Some require the decision-makers to participate in the weighting procedure and the values of the weights are fully dependent on their opinion. These methods belong to subjective weighting techniques characterized by uncertainty due to varying interpretations of the decision problem by different decision-makers [41]., i.e., AHP, Simultaneous Evaluation of Criteria and Alternatives (SECA) [42], Simple Multi-Attribute Rating Techniques (SMART) [43], etc. To overcome the disadvantages of subjective weighting techniques, various objective weighting techniques were proposed and widely used because they do not need expert knowledge of the problem anymore and are readily applied. The weight value calculated by the objective weighting techniques is only dependent on the inherent information and the mathematical equations. The objective weighting techniques include the entropy method, standard deviation method, statistical variance method, mean method, etc. [44]. As suggested by Duan, et al. [45], we use the entropy method to calculate the weight of each index, which effectively eliminates the influence of manual intervention and makes the results of the evaluation more objective and accurate.

Fuzzy set theory has been approved to be an effective approach to deal with uncertainty and ambiguity in MCDA [46–48]. Therefore, the integration of fuzzy set theory and MCDA methods would perfectly solve the ambiguous group decision problems. Previous studies have shown that the Fuzzy TOPSIS was widely used in various issues in recent decades. Bae, et al. [49] evaluated the health vulnerability caused by climate and air pollution in Korea by using the fuzzy TOPSIS. Rahim, et al. [50] showed the possibility of fuzzy logic utilization in assessing safety, health, and environmental risk and proposed a methodology based on the fuzzy-TOPSIS MCDA model for material selection suitable for the manufacturing sector. Milad Shafii, et al. [51] used fuzzy TOPSIS and fuzzy AHP methods to assess the performance of hospital managers in the hospitals owned by the Iranian Ministry of Health. Zhao, et al. [52] applied the fuzzy TOPSIS approach to evaluate the performance of Strong Smart Grid in China. From the above-mentioned studies, we can see that the method of fuzzy TOPSIS was more applicable and reliable than the traditional TOPSIS method in MCDA.

## Data and methods

**Fig. 1:**
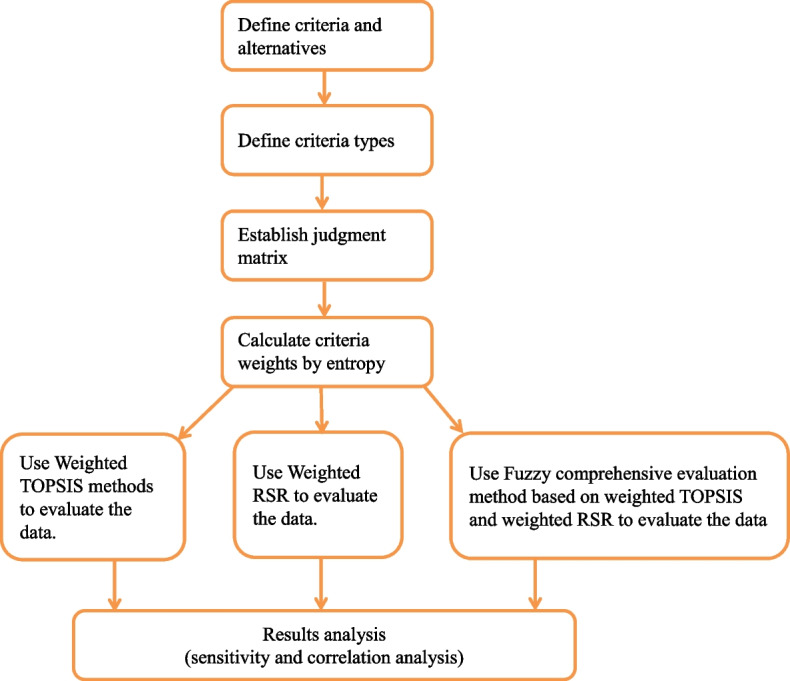
The flowcharts in this study

Figure 1 shows the MCDA problem-solving flowchart of this study.

### Patient and public involvement

No patient was involved in this study since it’s a kind of research on the statistical analysis of data, and all the data used in the study was publicly available in the China Health Statistics Yearbook.

### Data

The data construction and collection in the China Health Statics Yearbook is mainly responsed by the National Health Commission of the People’s Republic of China. And China Health Statistics Yearbook is publicly released by National Health Commission of the People’s Republic of China every year. Each Yearbook records the latest 10 years of CHC data. The data for this study (2000-2020 CHC data ) was sourced from the China Health Statistics Yearbook 2010-2021. According to the Yearbook’s guidance and relevant studies from Chinese researchers, a total of 6 criteria I_1_~I_6_ that directly reflect the work quality of CHC were selected as evaluation indicators to comprehensively evaluate the status of CHC in China from the year 2011 to 2020, which are I_1_**:** the percentage of low birthweight newborns (less than 2,500 gram, %), I_2_**:** perinatal mortality (‰), I_3_**:** the prevalence of low weight in children under 5 years old (%), I_4_**:** Neonatal visit rate (%), I_5_**:** systematic management rate of children under 3 years old (%), I_6_**:** systematic management rate of children under 7 years old (%). Table 1 shows the original data of CHC in China from 2000 to 2020.

**Table 1:** The original data on child health care in China from 2000 to 2020

**Year**	**I**_**1**_	**I**_**2**_	**I**_**3**_	**I**_**4**_	**I**_**5**_	**I**_**6**_
**2000**	2.40	13.99	3.09	85.8	73.8	73.4
**2001**	2.35	13.28	3.01	86.7	74.7	74.5
**2002**	2.39	12.47	2.83	86.1	73.9	74.0
**2003**	2.26	12.24	2.70	84.7	72.8	72.7
**2004**	2.20	11.08	2.56	85.0	73.7	74.1
**2005**	2.21	10.27	2.34	85.0	73.9	74.8
**2006**	2.22	9.68	2.10	84.7	73.9	75.0
**2007**	2.26	8.71	2.02	85.6	74.4	75.9
**2008**	2.35	8.74	1.92	85.4	75.0	77.4
**2009**	2.40	7.70	1.71	87.1	77.2	80.0
**2010**	2.34	7.02	1.55	89.6	81.5	83.4
**2011**	2.33	6.32	1.51	90.6	84.6	85.8
**2012**	2.38	5.89	1.44	91.8	87.0	88.9
**2013**	2.44	5.53	1.37	93.2	89.0	90.7
**2014**	2.61	5.37	1.48	93.6	89.8	91.3
**2015**	2.64	4.99	1.49	94.3	90.7	92.1
**2016**	2.73	5.05	1.44	94.6	91.1	92.4
**2017**	2.88	4.58	1.40	93.9	91.1	92.6
**2018**	3.13	4.26	1.43	93.7	91.2	92.7
**2019**	3.24	4.02	1.37	94.1	91.9	93.6
**2020**	3.25	4.14	1.19	95.5	92.9	94.3

(I_1_**:** the percentage of low birthweight newborns (less than 2,500 gram, %), I_2_**:** perinatal mortality (‰), I_3_**:** the prevalence of low weight in children under 5 years old (%), I_4_**:** Neonatal visit rate (%), I_5_**:** systematic management rate of children under 3 years old (%), I_6_**:** systematic management rate of children under 7 years old (%))

### Methods

#### Using the entropy weight method to determine each indicator’s weight

The entropy weight method, a method to determine the weights of indicators by evaluating the values of indicators under objective conditions [53], was recommended in our study to calculate each indicator’s weight according to the following 4 steps.

- Step 1: Establishing the judgment matrix

According to Table 1, we establish the following judgment matrix A :

$$\begin{array}{cc}\mathrm{A}=({a}_{ij}{)}_{m,n}& (\mathrm{i}=\mathrm{1,2},...,\mathrm{m};\mathrm{ j}=\mathrm{1,2},...\mathrm{n})\end{array}$$

$$(\mathrm{A}={a}_{ij}{)}_{m,n}=\left[\begin{array}{ccc}{a}_{11}& {a}_{12}& \begin{array}{cc}\cdots & {a}_{1n}\end{array}\\ {a}_{21}& {a}_{22}& \begin{array}{cc}\cdots & {a}_{2n}\end{array}\\ \begin{array}{c}\vdots \\ \begin{array}{c}{a}_{i1}\\ \vdots \\ {a}_{m1}\end{array}\end{array}& \begin{array}{c}\vdots \\ \cdots \\ \begin{array}{c}\vdots \\ {a}_{m2}\end{array}\end{array}& \begin{array}{cc}\begin{array}{c}\vdots \\ \begin{array}{c}{a}_{ij}\\ \vdots \\ {a}_{m3}\end{array}\end{array}& \begin{array}{c}\vdots \\ \begin{array}{c}\cdots \\ \vdots \\ {a}_{mn}\end{array}\end{array}\end{array}\end{array}\right]$$

In which m=21, *n*=6, $\mathrm{and\;}{a}_{ij}$ represent the *j*-th indicator’s value in the *i*-th year.

- Step 2: Normalize the judgment matrix.

The criteria are generally classified into 2 types: benefit and cost. The benefit criteria mean that the higher the value it is the better the result would be, while the cost criteria is valid the opposite. In our study, I_1_, I_2,_ and I_3_ are cost criteria, while others are benefit criteria. Because the higher the value of indicator I_1_ (the percentage of low birthweight newborns), indicator I_2_ (perinatal mortality), and indicator I_3_ (the prevalence of low weight in children under 5 years old), the worse the CHC status. Meanwhile, the higher the value of indicator I_4_ **(**Neonatal visit rate), I_5_ **(**systematic management rate of children under 3 years old), I_6_** (**systematic management rate of children under 7 years old), the worse the CHC status. We use Equations (1) and (2) to normalize the benefit criteria and cost criteria values, respectively.

1 $${S}_{ij}=\frac{{a}_{ij}-\mathrm{min}\{{a}_{ij}\}}{\mathrm{max}\left\{{a}_{ij}\right\}-\mathrm{min}\{{a}_{ij}\}}$$

2 $${S}_{ij}=\frac{\mathrm{max}\left\{{a}_{ij}\right\}-{a}_{ij}}{\mathrm{max}\left\{{a}_{ij}\right\}-\mathrm{min}\{{a}_{ij}\}}$$

- Step 3: Calculating the indicator’s entropy

In an evaluation problem that has m evaluated object with n indicators, the entropy for the *j-*th indicator is calculated as the equation (3):

3 $$\begin{array}{cc}{E}_{j}=-k\sum_{i=1}^{m}{f}_{ij}\mathrm{ln}{f}_{ij}& \mathrm{i}=\mathrm{1,2},...,\mathrm{m};\mathrm{ and j}=\mathrm{1,2},...,\mathrm{n};\end{array}$$

Where,$${f}_{ij}=\frac{{S}_{ij}}{\sum_{i=1}^{m}Sij}$$ , $$\mathrm{k}=\frac{1}{\mathrm{ln}m}$$.

Among them, ${f}_{ij}$ is the characteristic proportion of the *i*-th object.

- Step 4: Calculating the entropy weight

The *j*th indicator’s entropy weight $({w}_{j})$ was then calculated based on the Equation (3)

4 $${w}_{j}=\frac{1-{E}_{j}}{n-\sum_{j=1}^{n}{E}_{j}}$$

#### Entropy-weighted TOPSIS evaluation method

The entropy-weighted TOPSIS evaluation model has been widely used in MCDA applications due to its objectiveness, rationality, and effectiveness. It is an effective MCDA method to evaluate the performance of alternatives through similarity with the ideal solution [54]. Its basic concept is that the chosen alternative should have the shortest distance from the ideal solution and the farthest from the negative-ideal solution [55]. The detailed processes of applying the entropy-weighted TOPSIS method are given below:

- Step 1: Build the co-trending decision matrix

TOPSIS method requires all the criteria should have the same type, which is benefit type or cost type, in other words, the decision matrix must be the co-trending matrix. Thus, we first convert all the cost indicators (I_1_, I_2,_ and I_3_) in Table 1 into the benefit indicators by replacing each cost indicator’s value with 100 minus it, respectively.

- Step 2: Normalize the co-trending matrix

The co-trending matrix was then normalized by Equation (5), which eliminated the influence of the different measurement units. Then, a normalized matrix R was established.

5 $$r_{ij}=a_{ij}/\sqrt{{\textstyle\sum_{i=1}^n}\;a_{ij}^2}$$

Where ${r}_{ij}$ represent the normalized value of *j*-*th* indicator’s value in the *i*-*th* year.

- Step 3: Build the normalized matrix of weight

We built the normalized matrix of weight X by Equation (6).

6 $${x}_{ij}={w}_{j}{\cdot r}_{ij}$$

Where i=1, 2,...,21, and j=1,2,...6.

Namely, each index ${r}_{ij}$ multiply its the corresponding weight ${w}_{j}$ which is calculated by the entropy weight method mentioned above. Then, a normalized matrix of weight X was obtained as below:

$$\mathrm{X}=\left[\begin{array}{ccc}{{w}_{1}r}_{11}& {{w}_{2}r}_{12}& \begin{array}{cc}\cdots & {{w}_{6}r}_{16}\end{array}\\ {{w}_{1}r}_{21}& {{w}_{2}r}_{22}& \begin{array}{cc}\cdots & {{w}_{6}r}_{26}\end{array}\\ \begin{array}{c}\vdots \\ {{w}_{1}r}_{211}\end{array}& \begin{array}{c}\vdots \\ {{w}_{2}r}_{212}\end{array}& \begin{array}{cc}\begin{array}{c}\vdots \\ \cdots \end{array}& \begin{array}{c}\vdots \\ {{w}_{6}r}_{216}\end{array}\end{array}\end{array}\right]$$

- Step 4: Identify the ideal solution A^+^ and negative-ideal solution A^-^

The positive-ideal solutional X^+^ and negative-ideal solution X^-^ were determined by matrix X as follows:

7 $${\mathrm{X}}^{+} =(\mathrm{max}\{{x}_{i1}\},\mathrm{ max}\{{x}_{i2}\},...\mathrm{ max}\{{x}_{i6}\})$$

8 $${\mathrm{X}}^{-} =(\mathrm{min}\{{x}_{i1}\},\mathrm{ min}\{{x}_{i2}\},...\mathrm{ min}\{{x}_{i6}\})$$

Where max{${x}_{ij}$} and min{${x}_{ij}$} means the max and min value in the *j-th* column, respectively.

- Step 5: Calculate Euclidean distance

We calculated Euclidean distance from X^+^ and X^-^ for each alternative ${x}_{i}$ , respectively as follows:

9 $${D}_{i}^{+}=\sqrt{\sum_{j=1}^{n}{(x}_{j}^{+}-{x}_{ij}}{)}^{2}$$

10 $${D}_{i}^{-}=\sqrt{\sum_{j=1}^{n}({{x}_{j}^{-}-x}_{ij}}{)}^{2}$$

Where ${D}_{i}^{+}$ are Euclidean distances between *i-th* objective and positive-ideal solution, and ${D}_{i}^{-}$ are Euclidean distances between *i-th* objective and negative-ideal solution.

- Step 6: Calculate the relative closeness coefficient

The relative closeness coefficient of *i-th* objective is calculated by using Equation (11) :

11 $${C}_{i}=\frac{{D}_{i}^{-}}{{D}_{i}^{+}+{D}_{i}^{-}}$$

Where 0 $\le {C}_{i}\le 1$, and the larger the ${C}_{i}$ value, the better the performance of CHC in that year. Then, we ranked all the objectives according to their ${C}_{i}$ values.

#### Entropy-weighted RSR evaluation method

Entropy-weighted RSR (WRSR) is another comprehensive evaluation method that uses a rank transformation to calculate dimensionless statistical indexes from the matrix. The distribution of WRSR can be explored by the parameter statistical method. Generally, the WRSR indicators range from 0 (worst) to 1 (best), which was used to assess the state of the subjective [56]. The detailed processes are given below:

- Step 1: Rank the indicators

We first rank all the indicators in Table 1 based on the rules that indicators of benefit type are ranked in ascending order while indicators of cost type are ranked in descending order.

- Step 2: Calculate the WRSR

We then calculate the WRSR of each evaluation object (i.e work quality of the CHC in a year) by equation (12).

12 $${WRSR}_{i}=\frac{1}{n}\sum_{j=1}^{m}{w}_{j}{S}_{ij}$$

Where ${S}_{ij}$ is the rank of CHC indicators in China from 2000 to 2021, i=1, 2, ...,21; *m=6,* which is the index number of CHC, and ${w}_{j}$ is the weight of *j-th* indicator.

- Step 3: Sort the objectives

The last step is sorting the objective according to the WRSR values. The greater the value of *WRSR*_*i*_ , the better the performance of CHC.

#### Fuzzy comprehensive evaluation method

The FCE method is an application of the fuzzy set theory to make a synthetic assessment in a fuzzy decision environment with multiple criteria [57]. The FCE method used in our study is given below:

- Step 1: Calculate the coefficient *C*_*i*_ and *WRSR*_*i*_

The coefficient *C*_*i*_ and *WRSR*_*i*_ of each alternative can be obtained by using the entropy-weighted TOPSIS and weighted RSR method, respectively.

- Step 2: Calculate the rank of each alternative based on the Fuzzy Set theory

The coefficient *C*_*i*_ and *WRSR*_*i*_ were substituted to the following formula:

13 $${W}_{1}{C}_{i}+{W}_{2}{WRSR}_{i}$$

Where *W*_*1*_:*W*_*2*_ is the weight ratio for *C*_*i*_ and *WRSR*_*i*_, respectively. According to the previous study applying fuzzy set theory to a comprehensive evaluation work [58], the weight ratio *W*_*1*_:*W*_*2*_ is set to 0.1:0.9, 0.5:0.5, and 0.9:0.1, respectively.

- Step 3: Rank the alternative comprehensively

Since the weight ratio has 3 sets of values (i.e., 0.1:0.9, 0.5:0.5, and 0.9:0.1), we ranked all the alternatives 3 times based on the result calculated by the formula (13), respectively. Correspondingly, each alternative has 3 orders and we selected the order that appeared most frequently as the comprehensive order of the alternative. The greater the value, the better the performance of CHC.

### Sensitivity analysis through criteria weight change

Sensitivity analysis is an effective method to observe variations in the final result that was caused by the changes in the model’s parameters. In our study, sensitivity analysis was conducted by changing each criterion’s weight according to the changing rate ${\delta }_{k}$. The designed scheme was also applied in the previous study [19]. Specially, supposing *W*_*i*_ changes to ${W}_{i}^{*}$, i=1,2,...,6 and ${W}_{i}^{*} is$ calculated by the Equations (14) and (15).

14 $${W}_{k}^{*}={\delta }_{k}{W}_{k}$$

15 $${\delta }_{k}=\frac{{\gamma }_{k}-{\gamma }_{k}{W}_{k}{\prime}}{1-{\gamma }_{k}{W}_{k}{\prime}}$$

Where *k*=1,2,...n ( *n*=6), ${\gamma }_{k}$=0.01, 0.03, 0.06, 0.1, 0.2, 0.5, 0.8,1.0, 1.3, 1.8, 2.1, 2.5, 3, 3.5,4 and 4.5.${\delta }_{k}$ is the changing rate of ${W}_{k}$. The variable ${\gamma }_{k}$ is defined as the unitary variation rate of the variation of ${W}_{k}$.

Since the sum of the 6 indicator’s weights should be equal to 1 when *W*_*k*_ changes ${W}_{k}^{*}$, other weights will also change, which was calculated as Equation (16).

16 $$\begin{array}{c}W_{1}^{'}=\frac{w_1}{w_1+w_2+\dots+W_k^\ast+\dots w_i}\\W_{2}^{'}=\frac{w_2}{w_1+w_2+\dots+W_k^\ast+\dots w_i}\\\begin{array}{c}W_{i}^{'}=\frac{W_k^\ast}{w_1+w_2+\dots+W_k^\ast+\dots w_i}\\W_{n}^{'}=\frac{w_n}{w_1+w_2+\dots+W_k^\ast+\dots w_i}\end{array}\end{array}$$

Where ${W}_{k}{\prime}$ is the *k-th* indicator’s weight after changing.

Taking *W*_*1*_ as an example, because the unit change rate ${\gamma }_{1}$ was designed to 16 different values, a total of 16 sets of changed weights ${W}_{k}^{*} and {W}_{k}{\prime}$ can be derived from the above formulas $.$ Correspondingly, *C*_*i*_ also has 16 changed values which will be analyzed further. These changes were all based on the variation of *W*_*1.*_ With the same algorithms, the recalculated *C*_*i*_ based on the variation of other weights (i.e., *W*_*2*_, *W*_*3*_*, W*_*4*_, *W*_*5*_, *W*_*6*_) can be obtained. All the calculations in our study were implemented in Matlab 2019b and Microsoft Excel 2010.

## Results

### The entropy weight for each indicator

Table 2 shows each indicator’s entropy weight values, which shows I_1_ has the maximum weight value of 0.1962, and I_3_ has the minimum weight value of 0.1448.

**Table 2:** Entropy weight of each indicator in this study

	**I**_**1**_	**I**_**2**_	**I**_**3**_	**I**_**4**_	**I**_**5**_	**I**_**6**_
**Entropy (** ${{\varvec{E}}}_{{\varvec{j}}}$**)**	0.8463	0.8755	0.8866	0.8645	0.8619	0.8818
**Weight (** ${{\varvec{w}}}_{{\varvec{j}}}$**)**	0.1962	0.1590	0.1448	0.1730	0.1763	0.1509

(I_1_**:** the percentage of low birthweight newborns (less than 2,500 gram, %), I_2_**:** perinatal mortality (‰), I_3_**:** the prevalence of low weight in children under 5 years old (%), I_4_**:** Neonatal visit rate (%), I_5_**:** systematic management rate of children under 3 years old (%), I_6_**:** systematic management rate of children under 7 years old (%); ${{\varvec{E}}}_{{\varvec{j}}}$ is the Entropy of I_j_; ${{\varvec{w}}}_{{\varvec{j}}}$ is the weight of I_j_; where j =1,2,...6)

### Evaluation results of CHC in China from 2000 to 2020 based on the entropy-weighted TOPSIS method

**Fig. 2:**
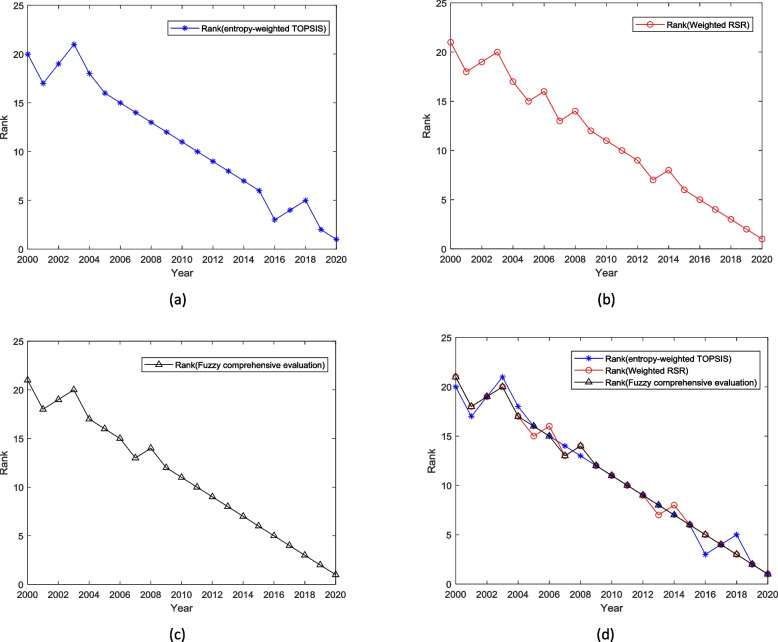
The ranks of child heal care in China from 2000 to 2020 with different methods. **a** the ranks calculated by the weighted TOPSIS method (**b**) the ranks calculated by the weighted RSR method (**c**) the ranks calculated by the Fuzzy comprehensive evaluation method (**d**) A comparison of the ranks calculated by the weighted TOPSIS method, weighted RSR method, and Fuzzy comprehensive evaluation method

The work quality of CHC in China from 2000 to 2020 was ranked based on the value of relative closeness coefficient *C*_*i*_ calculated by the entropy-weighted TOPSIS methods, the results are shown in Table 3 and Fig. 2a. The positive-ideal solutional X^+^ and negative-ideal solution X^-^ in this study were (0.0425, 0.0324, 0.0312, 0.0357, 0.0340, 0.0286) and (0.0429,0.0361,0.0318,0.0402,0.0433, 0.0371), respectively.

**Table 3:** Evaluation results of child health care in China from 2000 to 2020 based on the entropy-weighted TOPSIS method

**Years**	${{\varvec{D}}}_{{\varvec{i}}}^{+}$	${{\varvec{D}}}_{{\varvec{i}}}^{-}$	${{\varvec{C}}}_{{\varvec{i}}}$	**Rank**
2000	0.0133	0.0008	0.0569	20
2001	0.0126	0.0015	0.1058	17
2002	0.0130	0.0012	0.0822	19
2003	0.0138	0.0008	0.0547	21
2004	0.0130	0.0014	0.0964	18
2005	0.0127	0.0018	0.1228	16
2006	0.0127	0.0020	0.1363	15
2007	0.0121	0.0026	0.1740	14
2008	0.0116	0.0030	0.2029	13
2009	0.0100	0.0044	0.3062	12
2010	0.0074	0.0068	0.4789	11
2011	0.0056	0.0085	0.6027	10
2012	0.0039	0.0102	0.7238	9
2013	0.0026	0.0114	0.8166	8
2014	0.0021	0.0119	0.8497	7
2015	0.0015	0.0125	0.8931	6
2016	0.0013	0.0127	0.9091	3
2017	0.0013	0.0127	0.9060	4
2018	0.0013	0.0128	0.9055	5
2019	0.0009	0.0133	0.9351	2
2020	0.0005	0.0140	0.9679	1

**(** ${D}_{i}^{+}$ is Euclidean distance between *i* year CHC performance and positive-ideal CHC performance, ${D}_{i}^{-}$ are Euclidean distances between* i* year CHC performance and negative-ideal CHC performance, and ${C}_{i}$ is the relative closeness coefficient of *i-th* year CHC performance, where i =2000, 2001, ... 2020)

### Evaluation results of CHC in China from 2000 to 2020 based on the weighted RSR evaluation method

Then, we ranked the work quality of CHC in China from 2000 to 2020 based on the *WRSR*_*i*_ value calculated by the weighted RSR evaluation methods, the results are shown in Table 4 and Fig. 2b.

**Table 4:** Evaluation results of child health care in China from 2000 to 2020 based on the weighted RSR method

**Years**	**I**_**1**_	**rank**	**I**_**2**_	**rank**	**I**_**3**_	**rank**	**I**_**4**_	**rank**	**I**_**5**_	**rank**	**I**_**6**_	**rank**	***WRSR***_***i***_	**rank**
2000	2.4	9.5	13.99	1	3.09	1	85.8	7	73.8	3	73.4	2	0.2806	21
2001	2.35	13.5	13.28	2	3.01	2	86.7	9	74.7	8	74.5	5	0.4651	18
2002	2.39	11	12.47	3	2.83	3	86.1	8	73.9	5	74	3	0.3858	19
2003	2.26	17.5	12.24	4	2.7	4	84.7	1.5	72.8	1	72.7	1	0.3490	20
2004	2.2	21	11.08	5	2.56	5	85	3.5	73.7	2	74.1	4	0.4800	17
2005	2.21	20	10.27	6	2.34	6	85	3.5	73.9	5	74.8	6	0.5425	15
2006	2.22	19	9.68	7	2.1	7	84.7	1.5	73.9	5	75	7	0.5367	16
2007	2.26	17.5	8.71	9	2.02	8	85.6	6	74.4	7	75.9	8	0.6334	13
2008	2.35	13.5	8.74	8	1.92	9	85.4	5	75	9	77.4	9	0.6021	14
2009	2.4	9.5	7.7	10	1.71	10	87.1	10	77.2	10	80	10	0.6601	12
2010	2.34	15	7.02	11	1.55	11	89.6	11	81.5	11	83.4	11	0.7856	11
2011	2.33	16	6.32	12	1.51	12	90.6	12	84.6	12	85.8	12	0.8523	10
2012	2.38	12	5.89	13	1.44	15.5	91.8	13	87	13	88.9	13	0.8777	9
2013	2.44	8	5.53	14	1.37	19.5	93.2	14	89	14	90.7	14	0.9079	7
2014	2.61	7	5.37	15	1.48	14	93.6	15	89.8	15	91.3	15	0.8857	8
2015	2.64	6	4.99	17	1.49	13	94.3	19	90.7	16	92.1	16	0.9521	6
2016	2.73	5	5.05	16	1.44	15.5	94.6	20	91.1	17.5	92.4	17	0.9918	5
2017	2.88	4	4.58	18	1.4	18	93.9	17	91.1	17.5	92.6	18	0.9995	4
2018	3.13	3	4.26	19	1.43	17	93.7	16	91.2	19	92.7	19	1.0035	3
2019	3.24	2	4.02	21	1.37	19.5	94.1	18	91.9	20	93.6	20	1.0807	2
2020	3.25	1	4.14	20	1.19	21	95.5	21	92.9	21	94.3	21	1.1279	1

(I_1_: the percentage of low birthweight newborns (less than 2,500 gram, %), I_2_: perinatal mortality (‰), I_3_: the prevalence of low weight in children under 5 years old (%), I_4_: Neonatal visit rate (%), I_5_: systematic management rate of children under 3 years old (%), I_6_: systematic management rate of children under 7 years old (%);*WRSR*_*i*_ is Entropy-weighted rank-sum ratio of i-th year’s CHC performance in China, where i= 2000, 2001, .... 2020)

### Evaluation results of CHC in China from 2000 to 2020 based on the FCE method

Table 5 and Fig. 2c shows the detailed evaluation results of CHC in China from 2000 to 2020 based on the FCE method.

**Table 5:** Evaluation results of child health care in China from 2000 to 2020 based on the fuzzy comprehensive evaluation method

**Year**	**0.1* WRSR***_***i***_** +0.9** ${{\varvec{C}}}_{{\varvec{i}}}$	**rank**	**0.5WRSRi+0.5** ${{\varvec{C}}}_{{\varvec{i}}}$	**rank**	**0.9WRSRi+0.1** ${{\varvec{C}}}_{{\varvec{i}}}$	**rank**	**Fuzzy comprehensive rank**
2000	0.0793	21	0.1688	21	0.2582	21	21
2001	0.1417	17	0.2855	18	0.4292	18	18
2002	0.1126	19	0.2340	19	0.3554	19	19
2003	0.0841	20	0.2019	20	0.3196	20	20
2004	0.1348	18	0.2882	17	0.4416	17	17
2005	0.1648	16	0.3327	16	0.5005	15	16
2006	0.1763	15	0.3365	15	0.4967	16	15
2007	0.2199	14	0.4037	13	0.5875	13	13
2008	0.2428	13	0.4025	14	0.5622	14	14
2009	0.3416	12	0.4832	12	0.6247	12	12
2010	0.5096	11	0.6323	11	0.7549	11	11
2011	0.6277	10	0.7275	10	0.8273	10	10
2012	0.7392	9	0.8008	9	0.8623	9	9
2013	0.8257	8	0.8623	8	0.8988	7	8
2014	0.8533	7	0.8677	7	0.8821	8	7
2015	0.8990	6	0.9226	6	0.9462	6	6
2016	0.9174	3	0.9505	5	0.9835	5	5
2017	0.9154	4	0.9528	4	0.9902	4	4
2018	0.9153	5	0.9545	3	0.9937	3	3
2019	0.9497	2	1.0079	2	1.0661	2	2
2020	0.9839	1	1.0479	1	1.1119	1	1

(*WRSR*_*i*_ is Entropy-weighted rank-sum ratio of i-th year’s CHC performance, and ${C}_{i}$ is the relative closeness coefficient of *i-th* year CHC performance, where i= 2000, 2001, .... 2020)

The results indicate that all 3 evaluation methods above have approximately similar results which are that the top 5 performances of CHC were achieved in the latest 5 years (2016-2020) and the bottom 5 performances of CHC were achieved in the first 5 years (2000-2004). Figure 2d indicates that the trend of CHC performance in recent years is consistent. Generally, CHC in China improved year by year after 2000.

### Correlation analysis

**Fig. 3:**
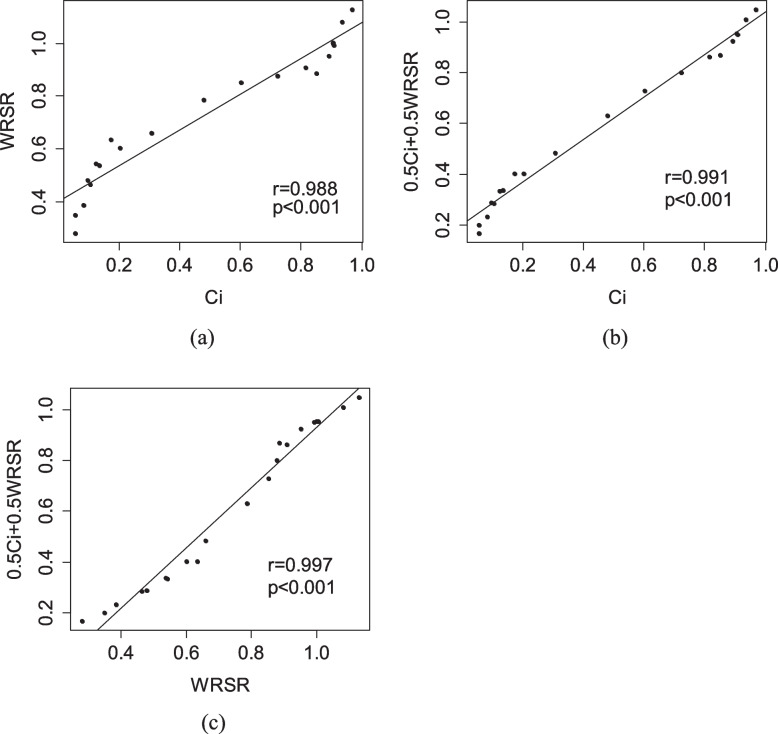
Spearman rank correlation analysis for the results calculated by the different methods. **a** Correlation between *C*_*i*_ and *WRSR* (**b**) orrelation between *C*_*i*_ and 0.5 *C*_*i*_* +*0.5*WRSR* (**c**) Correlation between *WRSR* and 0.5 *C*_*i*_*+*0.5*WRSR. WRSR* is the Entropy-weighted rank-sum ratio of the CHC performance in i-th year, *C*_*i*_ is the relative closeness coefficient of the CHC performance in i-th year, where i=2000,2001,...2020

The correlation of different evaluation results (i.e., weighted TOPSIS method, WRSR, and FCE) was then analyzed by Spearman’s rank correlation coefficient. Figure 3a-c shows the correlation between *C*_*i*_ and *WRSR*_*i*_, the correlation between *C*_*i*_ and 0.5*C*_*i*_ +0.5*WRSR*_*i*_, and the correlation between *WRSR*_*i*_ and 0.5*C*_*i*_ +0.5*WRSR*_*i,*_ respectively. The calculated coefficients of Spearman’s rank correlation indicate all these correlations are significantly positively correlated.

### Sensitivity analysis

**Fig. 4:**
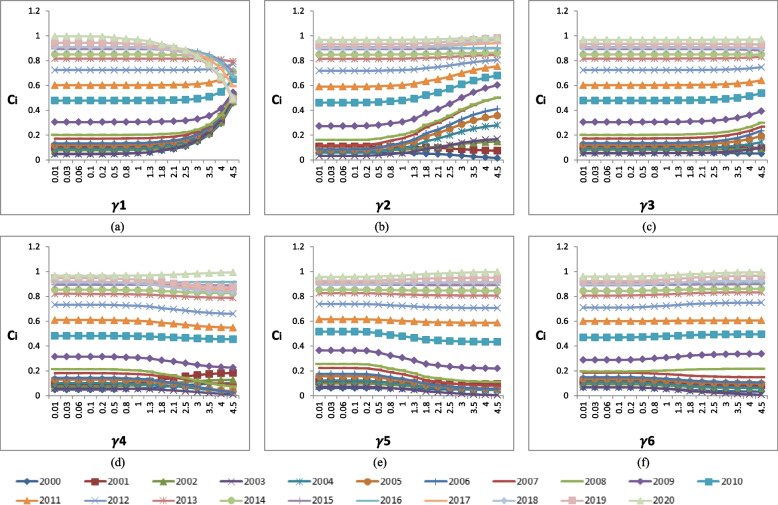
Weight of *C*_*i*_ sensitivity analysis. *C*_*i*_ is the relative closeness coefficient of *i-th* year CHC performance, where i= 2000, 2001, 2020. **a**-**f** is the *C*_*i*_ value under different unitary variation ratio γγ_*kk*_ for W1 - W6, respectively, where k=1,2,. 16

Table 6 and Fig. 4a show the changing weights of each indicator under the different unitary variation ratio ${\gamma }_{1}$ for W1. With the same method, the changing weights under different unitary variation ratio ${\gamma }_{k}$ for W_2_, W_3_, W_4_, W_5_, and W_6_ can be obtained, which are shown in Fig. 4b-f. The original ranking calculated by entropy-weight TOPSIS for each year’s CHC performance is the ranking when ${\gamma }_{k}=1$.

**Table 6:** The weights calculated by the different unitary variation ratios

Unitary variation ratio ${\gamma }_{k}$	${\boldsymbol W}_{\mathbf1}\boldsymbol'$	${\boldsymbol W}_{\mathbf2}\boldsymbol'$	${\boldsymbol W}_{\mathbf3}\boldsymbol'$	${\boldsymbol W}_{\mathbf4}\boldsymbol'$	${\boldsymbol W}_{\mathbf5}\boldsymbol'$	${\boldsymbol W}_{\mathbf6}\boldsymbol'$
0.01	0.0020	0.1974	0.1797	0.2148	0.2188	0.1873
0.03	0.0059	0.1966	0.1790	0.2139	0.2180	0.1866
0.06	0.0118	0.1954	0.1780	0.2126	0.2167	0.1855
0.1	0.0196	0.1939	0.1766	0.2110	0.2150	0.1840
0.2	0.0392	0.1900	0.1730	0.2067	0.2107	0.1803
0.5	0.0981	0.1784	0.1624	0.1941	0.1978	0.1693
0.8	0.1569	0.1667	0.1518	0.1814	0.1849	0.1582
1	0.1962	0.1590	0.1448	0.1730	0.1763	0.1509
1.3	0.2550	0.1473	0.1342	0.1603	0.1634	0.1398
1.8	0.3531	0.1279	0.1165	0.1392	0.1419	0.1214
2.1	0.4120	0.1163	0.1059	0.1265	0.1289	0.1104
2.5	0.4904	0.1008	0.0918	0.1096	0.1117	0.0956
3	0.5885	0.0814	0.0741	0.0885	0.0902	0.0772
3.5	0.6866	0.0620	0.0564	0.0674	0.0687	0.0588
4	0.7848	0.0426	0.0388	0.0463	0.0472	0.0404
4.5	0.8829	0.0232	0.0211	0.0252	0.0257	0.0220

(${\gamma }_{k}$ is the different unitary variation ratio for, where k=1,2,...16; ${W}_{j}{\prime}$ is the weight of j-th indicator calculated by the ${\gamma }_{k}$, where j=1,2,...6 )

## Discussion

### Evaluation methods

Three different methods of weighted TOPSIS, weighted RSR, and FCE were applied in this study to evaluate the CHC work in China from 2000 to 2020. Each of them has its characteristics. The advantages of the weighted TOPSIS method are ease of application, universality, and consideration of distances to an ideal solution. Its disadvantages are low sensitivity and sensitivity to the interference of outliers [59]. A significant advantage of the weighted RSR method is that the interference of outliers is limited because the rank of original data has been used. Meanwhile, the disadvantage is that some potentially useful information is lost [60]. To overcome the disadvantages of weighted TOPSIS method and weighted RSR method, the FCE method has been used in this study, making the results more effective and reliable. The final evaluation result of FCE is determined by the most frequently appearing results in the designed schemes, reflecting the overall changing trend of the results [58]. Due to the above reasons, we recommend the FCE method to synthetically evaluate the CHC in China from 2000 to 2020.

### Evaluation results analysis

The results of the weighted TOPSIS method based on *C*_*i*_ value show that CHC in China improved year by year from 2003 to 2016 and a small downward trend appeared during 2001- 2003 (Fig. 2a, Table 3) which also appeared in the results of the weighted RSR method and FCE method, indicating the CHC in China from 2001 to 2003 maybe not as well as other years’ performance. Besides, slight declines appeared in 2005 and 2006, 2007 and 2008, 2013, and 2014 in the results of the weighted RSRS method, shown in Fig. 2b and Table 4. As for the results of FCE (Fig. 2c, Table 5), a slight decline appeared in 2007 and 2008. However, though some slight declines appeared, the overall trend of CHC in China from 2000 to 2020 is upward steadily without changing, as shown in Fig. 2d.

The weights used in the weighted TOPSIS method and weighted RSR methods were the entropy weights. It has the advantage of objectivity, making the results more objective and reliable. The entropy weights listed in Table 2 showed that the indicator of I_1_ (the percentage of low birthweight newborns) has a maximum weight value of 0.1962 and the minimum weight value of 0.1448 is for I_3_ (the prevalence of low weight in children under 5 years old). To observe the impact of weight variation on the final results, we performed the sensitivity analysis in the study, and the results are shown in Fig. 4. From Fig. 4a we can see that the *C*_*i*_ values gradually decrease when ${\gamma }_{k}$> 1.3 for the ranking of 2014-2020, and gradually increase when ${\gamma }_{k}$> 1.3 for the ranking of 2000-2009. The original ranking was disordered when ${\gamma }_{k}$> 3.5. Figure 4b shows that the ranking of each year’s CHC performance is not sensitive to the variation of W_2_ except for the year 2003. Figure 4c shows that the impact of W_3_ variation is limited on the ranking of each year’s CHC performance. Figure 4d shows that the variation of W_4_ has a certain impact on the ranking results in 2019, 2018, 2008,2007, 2001, and 2000. Figure 4e and f also show that each year’s CHC performance is not sensitive to the variation of W_5_ and W_6._ Besides, the results of Spearman rank correlation analysis indicated that the rank results of the weighted TOPSIS, the weighted RSR, and FCE are significantly positively correlated. In summary, the CHC in China from 2000 to 2020 improved year by year. This is inseparable from the efforts of all Chinese people and China's medical and health reform in recent decades.

### Effective policy

As early as 1992, the State Council of China issued the Planning Outline of Child Development in China in the 1990s, which was a national action plan for children to achieve their developmental potential, followed by the Child Development Outline of China (2001-2010) and Child Development Outline of China (2011-2020), which present national goals and strategies of 10-years plan for child development across health, child protection, education, environment, and social protection sectors [61]. The Chinese government made great progress in improving CHC work through legislation and investment. In 1994, the Law of the People’s Republic of China (PRC) on Maternal and Infant Health Care was enacted to guarantee the smooth implementation of policies for maternal and child health care [62]. Up to 2008, maternal and child health care hospitals or service centers have been established in every province, city, and county in China. Even township clinics have employed maternal and child health care staff [63]. The data of the China Health Statistic Yearbook 2003 and 2019 indicates that from 2003 to 2020 the numbers of health technicians, licensed physicians, and registered nurses in maternal and child health institutions in China have greatly increased from 145,610 to 428,809, from 59,340 to 136,820, from 40,476 to 196,000, with an average annual growth rate of 3.99%, 1.58%, and 8.24%, respectively. In 2009, the Chinese government launched an ambitious plan of health care system reform with the goal of providing universal coverage of essential health services for all Chinese citizens by 2020 and achieved substantial positive results that have even overtaken many developing countries [64]. The Chinese government also cooperated with international organizations to improve the CHC, such as World Health Organization (WHO), United Nations International Children's Fund (UNICEF), World Bank, etc. Such international conventions provided China with opportunities to develop a policy framework aimed at improving maternal and child health care work in China [65]. The China-UNICEF Integrated Early Child Development (IECD) in Poo Rural Regions Project was launched by the Government of China under support from UNICEF in 2013 [66]. Since the deepening of medical reform, China comprehensively implemented national basic public health service programs to freely provide 12 kinds of items including maternal and child health care services. The Chinese government announced the Health China 2030 blueprint in 2016, which aims to provide universal health security for all citizens by 2030 [67]. Besides, programs such as China’s Family Plan, Reinforcing Maternal and Child Health Care, Reinforcing Essential Health Service in Poor Rural Arear, Eliminating Newborn Tetanus in 1000 Counties in Midwestern Regions, etc, have been implemented successfully and achieved great success. The policies implemented in China may have little bias in different regions due to the different conditions and environments, however, the overall direction of policies is consistent. All in all, with the implementation of a series of effective policies and great efforts of Chinese people, the CHC in China improved year by year. 

### Limitation

This article only applied 3 evaluation methods on CHC in China, i.e. weighted TOPSIS, weighted RSR, and FCE methods. However, other classical evaluation methods, such as Grey Relational Analysis(GRE), Analytic Hierarchy Process(AHP), Data Envelopment Analysis (DEA), etc., were not applied. The evaluation methods in this article are not comprehensive enough. Meanwhile, due to the authors’ limited understanding and knowledge, some analyses in this article may be inaccurate and subjective. However, scientific methods for evaluating the CHC performance in China and theoretical support for future decision-makers are provided.

## Conclusion

In this study, we applied methods of the weighted TOPSIS, weighted RSR, and FCE methods to comprehensively evaluate the CHC status in China from 2000 to 2020. A total of 6 indicators were selected and each indicator’s weight was calculated by the entropy weight method objectively. FCE method, based on fuzzy theory and combining the results of the weighted TOPSIS methods and weighted RSR methods, was recommended since it effectively overcomes the disadvantages of a single evaluation method and the result is more reliable. Though the 3 rank results calculated by the different methods are not exactly the same, the overall trend was consistent, namely the CHC in China from 2000 to 2020 improved year by year, and the best CHC performance was achieved in 2020.

## Data Availability

Websit link: http://www.nhc.gov.cn/mohwsbwstjxxzx/tjzxtjcbw/tjsj_list.shtml. No additional data is available.
